# Corrosion of Low-Alloy Steel in Ethanolamine Steam Generator Chemistry—The Effect of Temperature and Flow Rate

**DOI:** 10.3390/molecules30020418

**Published:** 2025-01-20

**Authors:** Iva Betova, Martin Bojinov, Vasil Karastoyanov

**Affiliations:** 1 Institute of Electrochemistry and Energy Systems, Bulgarian Academy of Sciences, 1113 Sofia, Bulgaria; 2 Department of Physical Chemistry, University of Chemical Technology and Metallurgy, 8 Kliment Ohridski Blvd., 1756 Sofia, Bulgaria; martin@uctm.edu (M.B.); vasko_kar@uctm.edu (V.K.)

**Keywords:** ethanolamine, low-alloy steel, flow-assisted corrosion, electrochemical impedance spectroscopy, hydrodynamic calculations, kinetic model

## Abstract

The corrosion of low-alloy steel in ethanolamine solution, simulating steam generator chemistry, is studied by in situ chronopotentiometry and electrochemical impedance spectroscopy combined with ex situ analysis of the obtained oxide films and model calculations. Hydrodynamic calculations of the proposed setup to study flow-assisted corrosion demonstrate that turbulent conditions are achieved. Quantum chemical calculations indicate the adsorption orientation of ethanolamine on the oxide surface. Interpretation of impedance spectra with a kinetic approach based on the mixed-conduction model enabled estimating the rate constants of oxidation at the alloy–oxide interface, as well as charge transfer and ionic transport resistances of the corrosion process. In turbulent conditions, the dissolution of Fe oxide and ejection of Fe cations are enhanced, leading to Cr enrichment in the oxide and alteration of its electronic and electrochemical properties that influence the corrosion rate.

## 1. Introduction

Alkanolamines, such as monoethanolamine (MEA) [1,2] are used extensively for capture and storage of carbon originating from the combustion of flue gas [3], and also as alkalizing and passivating agents in steam generators of nuclear and conventional power plants [4,5,6,7]. Carbon capture by MEA has been studied in depth [8,9,10,11,12,13,14,15,16] and can potentially represent a source of its introduction into the environment as an emerging contaminant. MEA has been demonstrated to have passivating effects on corrosion in steam generator materials, particularly low-alloy steels, by reacting with corrosion layers [17]. In both of these contexts, the adsorption of MEA and its interaction with surface oxide films merit special attention.

Flow-assisted corrosion (FAC) [18,19,20,21,22,23] is an important phenomenon that controls the source term of soluble iron (in both ionic and colloidal form) that is transported through the system and deposited as sludge in critical areas such as tube support plates, causing deterioration of thermohydraulic properties and under-deposit corrosion due to accumulation of impurities. Several parameters influence FAC rates, viz. the geometrical configuration of the components, piping orientation, position inside the pipe, flow Reynolds number, water chemistry (i.e., the type of alkalizing agent, such as MEA), temperature, piping material (especially Cr content of steel), and flow turbulence structure [18,19,20]. Characterization of the thin iron oxide layers formed on carbon and low-alloy steel components under the hydrodynamical and chemical conditions prevailing within the secondary circuit of nuclear plants has been attempted in several papers [24,25,26,27,28], whereas studies of the FAC with electrochemical methods are comparatively scarce [29,30]. In particular, no in situ electrochemical impedance data during FAC or their quantitative interpretation with a kinetic model have been reported with the exception of a recent pre-study of ours [31].

The aim of the present study is to investigate the flow-assisted corrosion of a low-alloy steel containing mainly Cr and as little as possible of other alloying elements in MEA solution with a concentration typically used as steam generator coolant to ensure a room-temperature pH close to 10 (10–30 ppm, or 0.1–0.5 mmol dm^−3^ [6,7]) with a specially designed setup to ensure turbulent conditions. First, the structure and orientation of MEA molecule are obtained via quantum chemical calculations with its interaction with surface oxide in mind. Second, hydrodynamic calculations of the flow-through cell are described to demonstrate that turbulent conditions are achieved. Third, in situ chronopotentiometric and impedance spectroscopic measurements in the temperature range 130–230 °C are presented and discussed. The thickness and in-depth composition of oxides depending on temperature are estimated by Glow-Discharge Optical Emission Spectroscopy (GDOES). Quantitative interpretation of the impedance data with a kinetic model enables its parameterization in terms of interfacial rate constants, charge transfer and ion transport resistances, thickness of and field strength in the forming oxide depending on exposure time, temperature and flow rate. Finally, the model is validated by comparison with operational data for FAC and directions for future research are indicated.

## 2. Results

### 2.1. Quantum Mechanical Calculations of Ethanolamine Structure

**Figure 1:**
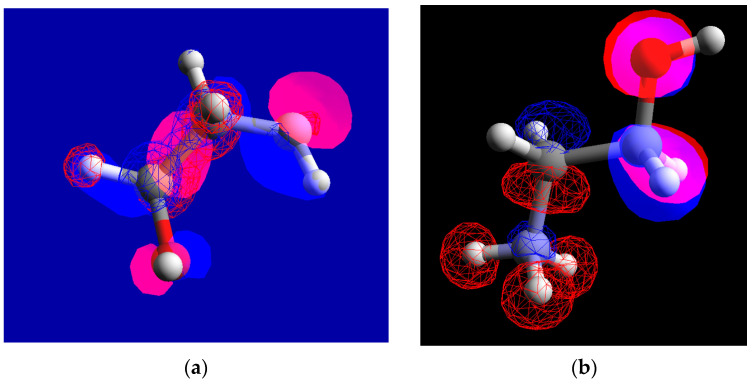
Structure of ethanolamine (**a**) and ethanol-ammonium cation (**b**). Highest occupied molecular orbitals (transparent) and lowest unoccupied orbitals (mesh) are shown. Negative charges indicated in blue and positive in red.

Depending on solution pH, MEA exists in mainly two forms—neutral (HOCH_2_CH_2_NH_2_) and cation (HOCH_2_CH_2_NH_3_^+^) [1,3]. At the pH of 10 employed in the present study, the neutral form amounts to 76% and the ionized form to 24%, according to calculations using the dissociation constant of MEA (pK_a_ = 9.5 [1]). Using the Method of Neglecting Differential Overlap (MNDO) implemented in open source software Arguslab 4.0.1, the structures of both forms were calculated. The highest occupied and the lowest unoccupied orbitals are presented in Figure 1. The semi-empirical MNDO method was chosen because it gives fast results for small molecules, whereas for interactions of molecules with surfaces, Density Functional Theory (DFT) is preferred. A molecular dynamics study of the interaction of MEA with magnetite surfaces will be presented and discussed in a companion paper.

As the pH of zero charge of corrosion layers on carbon and low-alloy steels is most probably close to that of magnetite (5.5–6.0 in the temperature range 130–230 °C [32]), it can be assumed that in the present conditions (alkaline pH at all studied temperatures), MEA adsorbs with the positively charged nitrogen, interacting with the negatively charged oxide surface. This adsorption would result in a compensation of charges at the oxide surface and suppression of electron charge transfer reactions, i.e., MEA would act as an inhibitor to the corrosion process not only by maintaining pH values that minimize oxide solubility, but also interfering in the corrosion reaction. This hypothesis remains, of course, to be verified by more detailed studies of the interaction of MEA with magnetite surfaces.

### 2.2. Evolution of Water Chemistry Parameters in Transition and Turbulent Regimes

**Figure 2:**
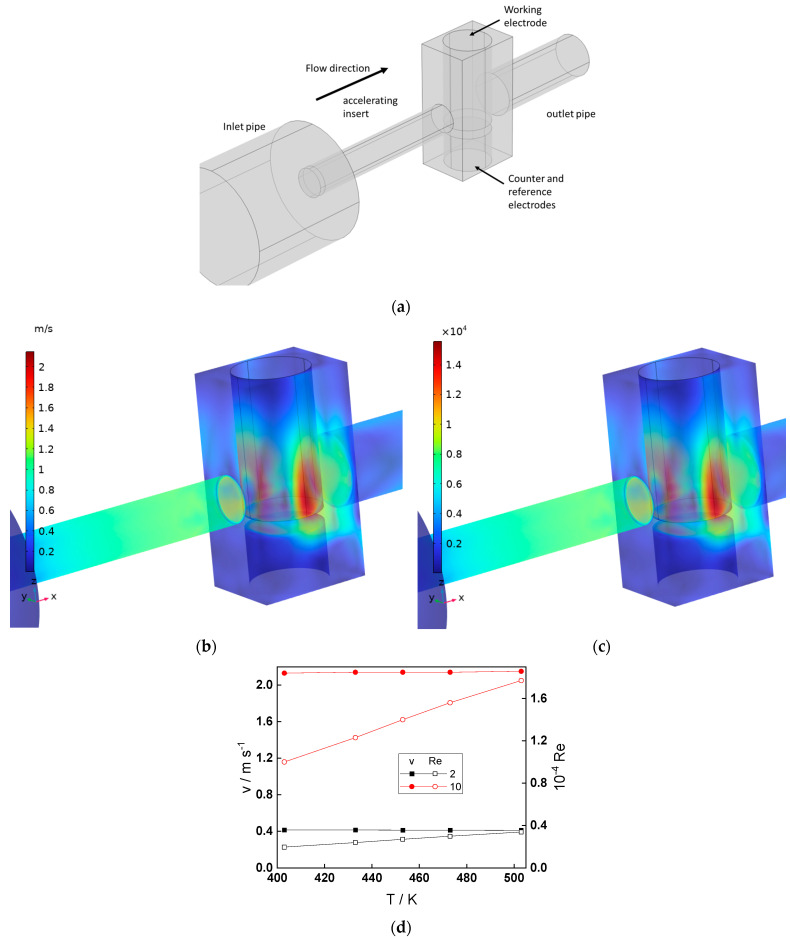
Schematic view of the flow-through cell (**a**) and hydrodynamic conditions: (**b**) 3-D distribution of the linear velocity at 230 °C, (**c**) 3-D distribution of the Reynolds number at 230 °C, (**d**) dependences of the linear fluid velocity and Reynolds number on temperature for two volume flow rates (in dm^3^ h^−1^).

The experimental setup used in the present investigation is illustrated in Figure 2a. The profiles of linear fluid velocity and Reynolds number in the flow-through cell calculated by the k-ε turbulent flow model are presented in Figure 2b,c. The largest velocities and Reynolds numbers are observed at the front and sides of the working electrode. The dependences of those two parameters on temperature and inlet volume flow rate are given in Figure 2d and indicate that turbulent conditions (Re > 10^4^) have been achieved in the whole temperature interval at an inlet volume flow rate of 10 dm^3^ h^−1^, whereas a transition regime (Re of the order of 2–4 × 10^3^) prevails at the lower flow rate (2 dm^3^ h^−1^).

**Figure 3:**
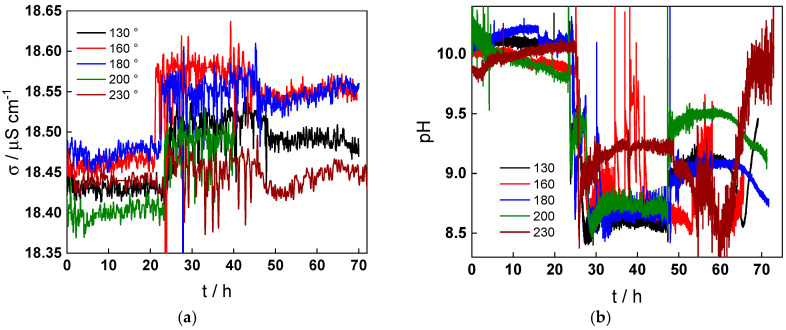
Evolution of specific conductivity (**a**) and pH (**b**) of the solution with time at all the studied temperatures. At t = 24 h, volume flow rate changed from 2 to 10 dm^3^ h^−1^, and at t = 48 h, back from 10 to 2 dm^3^ h^−1^.

The evolution of specific conductivity and pH at room temperature with time during typical experiments at all studied temperatures is presented in Figure 3a,b. When the inlet volume flow rate is increased from 2 to 10 dm^3^ h^−1^, an increase in conductivity and accordingly a decrease in pH is observed, indicating dissolution of the outer layer of oxide resulting in an increase in soluble iron concentration that leads to higher conductivity and lower pH. When the flow rate is changed back to 2 dm^3^ h^−1^ (at 48 h) at temperatures lower than 200 °C, conductivities do not return to their original values, i.e., the dissolution is most probably sustained. This hypothesis is corroborated by the fact that the pH in this temperature interval also does not return to the original value of 10. On the contrary, at higher temperatures, the effect of flow rate change on both conductivity and pH is on the overall smaller and reversible, indicating that the dissolution due to turbulent flow is a transient phenomenon.

### 2.3. Electrochemical Measurements

**Figure 4:**
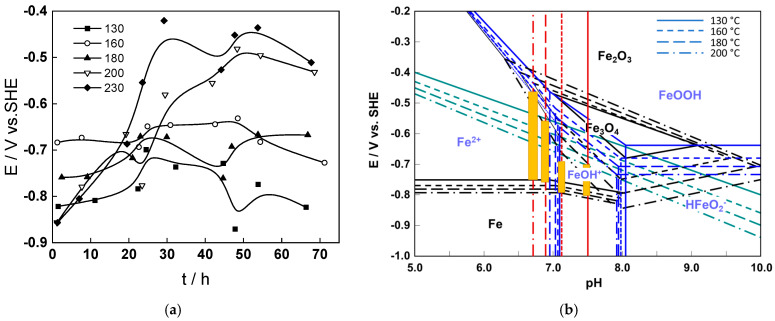
Corrosion potential vs. time at all the studied temperatures (**a**) and its values (vertical yellow bars) superimposed on the respective E-pH diagrams of the Fe-H_2_O system calculated at 130–200 °C (**b**). At t = 24 h, volume flow rate changed from 2 to 10 dm^3^ h^−1^, and at t = 48 h, from 10 to 2 dm^3^ h^−1^.

The observations from the water chemistry parameters are in qualitative accordance with the evolution of corrosion potential with time, Figure 4a. Changing the flow rate from 2 to 10 dm^3^ h^−1^ leads to an increase in the corrosion potential, but this increase is rather small at temperatures lower than 200 °C, whereas at 200 and 230 °C, a larger shift is observed, most probably related to a passivation process. This suggestion is confirmed by placement of the potential values in the respective E-pH diagrams of the Fe-H_2_O system (Figure 4b). Indeed, for temperatures lower than 200 °C, the potentials are situated in the region of divalent iron ion FeOH^+^, i.e., a dissolved product of corrosion is stable. Conversely. At higher temperatures, the potentials reach the magnetite (Fe_3_O_4_) stability region, i.e., passivation is more likely to occur. An attempt to further correlate these observations to the composition of the outermost layer of oxide is made below.

**Figure 5:**
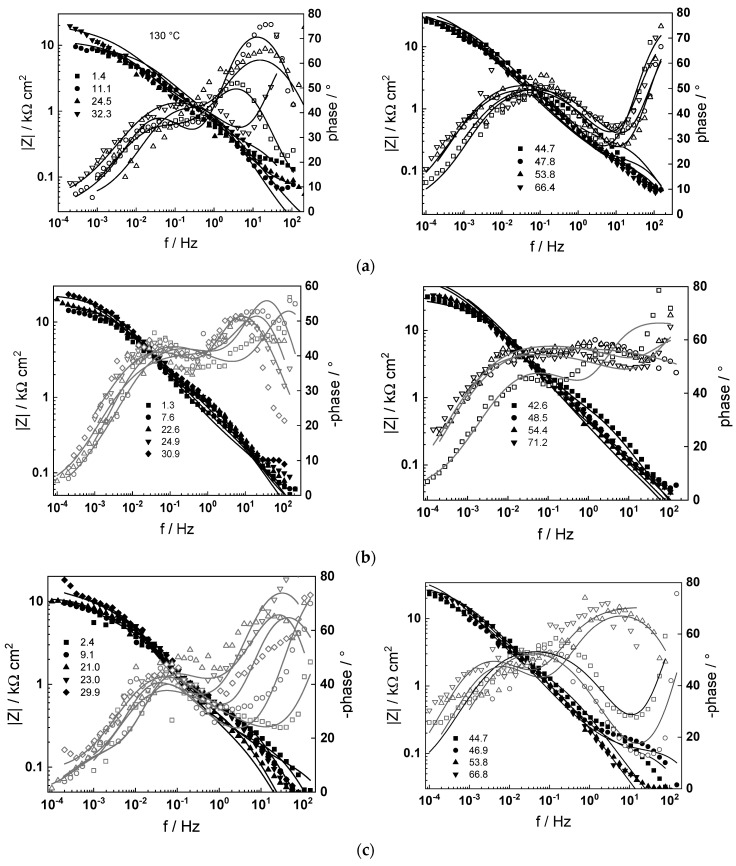
Electrochemical impedance spectra depending on time of exposure at 130 (**a**), 160 (**b**) and 180 °C (**c**). At t = 24 h, volume flow rate changed from 2 to 10 dm^3^ h^−1^, and at t = 48 h, from 10 to 2 dm^3^ h^−1^. Points—experimental values (full symbols—|Z|, open symbols—phase); lines—best-fit calculation according to the kinetic model.

**Figure 6:**
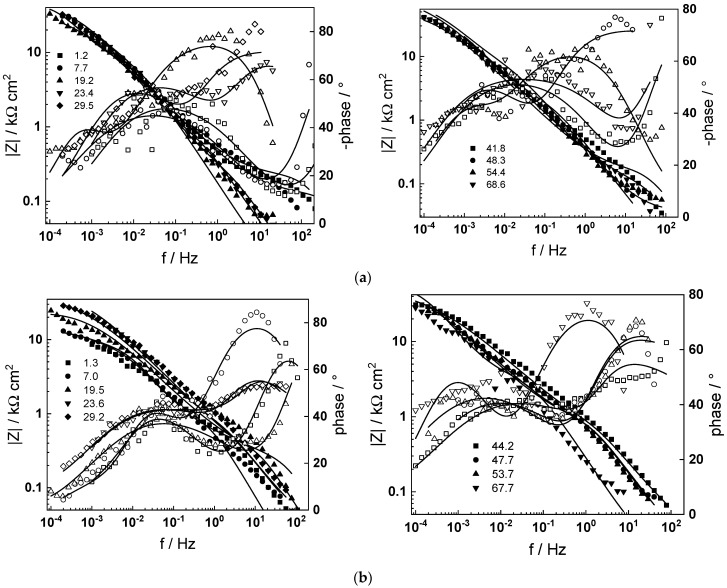
Electrochemical impedance spectra depending on time of exposure at 200 (**a**) and 230 °C (**b**). At t = 24 h, volume flow rate changed from 2 to 10 dm^3^ h^−1^, and at t = 48 h, from 10 to 2 dm^3^ h^−1^. Points—experimental values (full symbols—|Z|, open symbols—phase); lines—best-fit calculation according to the kinetic model.

**Figure 7:**
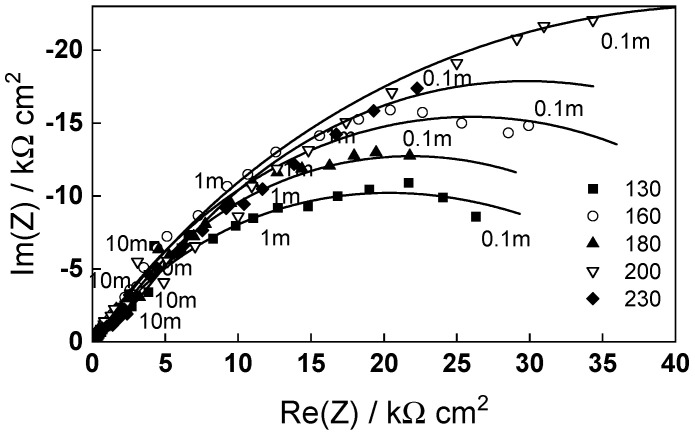
Electrochemical impedance spectra at t = 72 h depending on temperature. Parameter is frequency in Hz. Points—experimental values; lines—best-fit calculation according to the kinetic model.

The impedance spectra of low-alloy steel depending on time of exposure are shown in Figure 5 (130, 160 and 180 °C) and Figure 6 (200 and 230 °C), respectively, in Bode coordinates. To facilitate identification of processes at high frequencies, 95% of the ohmic resistance of the electrolyte was subtracted from the spectra. In general, the magnitude of the impedance at low frequencies (approximating the polarization resistance of corrosion) increases with time and reaches stable values after 40–50 h of exposure, the effect of changing the flow rate being comparatively insignificant, which indicates that processes of ion transport in bulk solution are not detected and therefore not rate-determining steps for corrosion. The dependence of the impedance spectra after ca. 72 h of exposure on temperature is presented in Figure 7 in complex plane coordinates. In analogy to our previous paper on carbon steel [31], the evolution of polarization resistance (approximated with the diameter of the semicircle) with temperature is not monotonous, the values being the lowest at 130 °C, larger at 160 and again lower at 180 °C. The resistances at 200 and 230 °C are considerably larger, indicating a more stable passivation.

Preliminary analysis indicates three processes with different time constants in the phase angle-frequency dependences. Following previous interpretation of analogous measurements of engineering metals in high-temperature aqueous solutions [31,33,34], the process with the highest characteristic frequency is assigned to electrical properties of the corrosion film, and hence to the electron migration step of the corrosion reaction. In turn, the constant at intermediate frequencies corresponds to the interfacial electron transfer step of corrosion, expressed by the charge transfer resistance in parallel with the interfacial capacitance. Finally, the low-frequency process is identified as the transport of ionic point defects in the oxide during corrosion. A further quantitative insight in the relative importance of the processes identified with these time constants will be given in the Section 3 owed to the quantitative comparison of the data with a pertinent kinetic model.

### 2.4. Oxide Thickness and Composition

**Figure 8:**
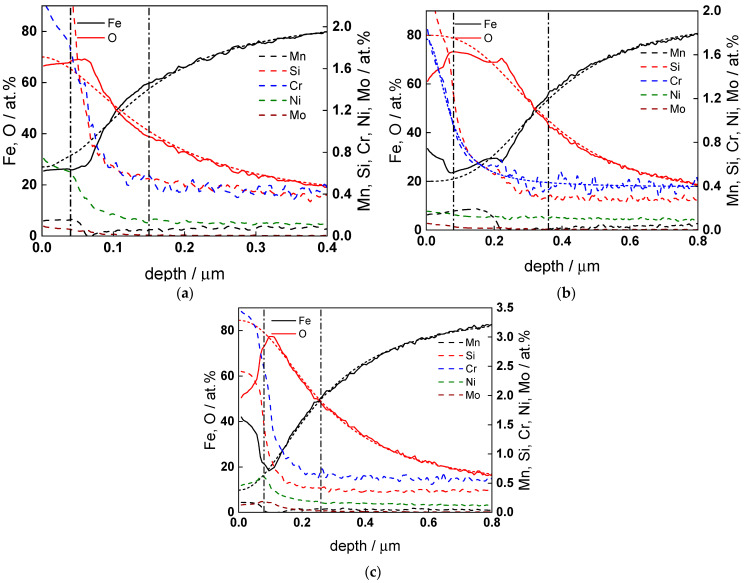
GDOES depth profiles of atomic concentrations of constituent elements for the low-alloy steel after 72 h of exposure at 130 (**a**), 160 (**b**) and 180 °C (**c**). Vertical lines show oxide layer–alloy and contamination layer–oxide layer interfaces estimated by sigmoidal fitting of oxygen and iron profiles (shown with short dash lines).

**Figure 9:**
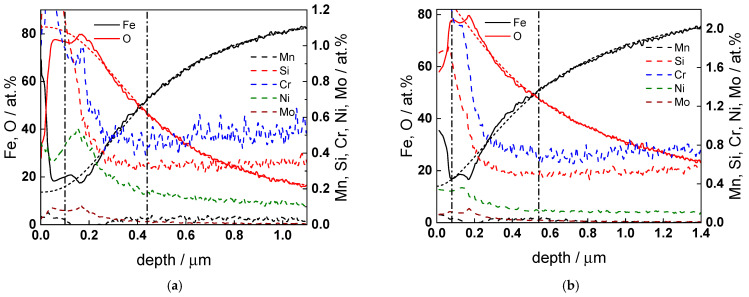
GDOES depth profiles of the atomic concentrations of constituent elements for the low-alloy steel after 72 h of exposure at 200 (**a**), and 230 °C (**b**). Vertical lines show oxide layer–alloy and contamination layer–oxide layer interfaces estimated by sigmoidal fitting of oxygen and iron profiles (shown with short dash lines).

GDOES depth profiles of the oxides after 72 h of exposure at all temperatures are collected in Figure 8 and Figure 9. A thin contamination layer containing carbon and nitrogen that is ubiquitously formed during transfer of samples to the analysis chamber is observed at all temperatures. It is followed by an inner layer with a composition close to magnetite. A considerable enrichment of Cr (with a factor of 10–20 when normalized to total cation content) is found in the outermost part of the oxide layer, accompanied by a lower, but detectable enrichment of Ni and possibly Mn and Mo (the uncertainty in atomic concentration of these two constituents is of course much larger due to their very low content in both the oxide and the matrix). In particular, the enrichment of Cr at the oxide–coolant interface plays an important role since its presence leads to a decrease in both oxide dissolution and cation ejection rates that ultimately affect the extent of corrosion.

**Figure 10:**
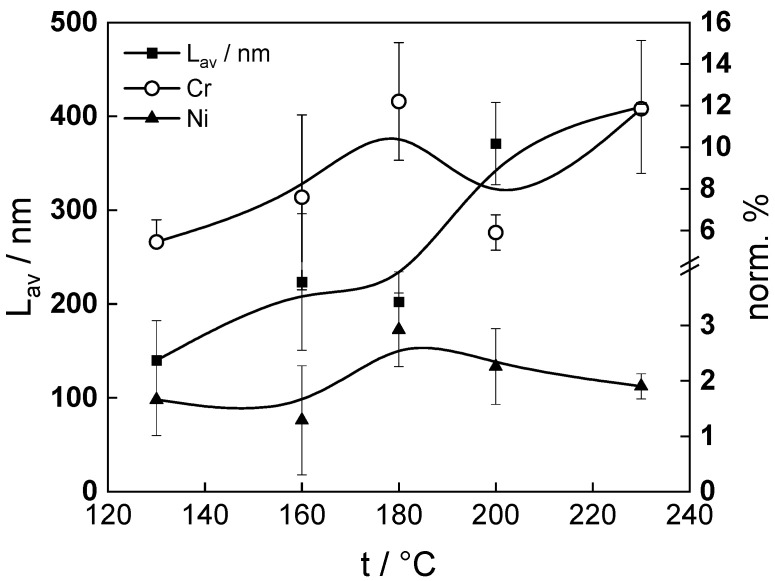
Average thickness of the oxide layer (left axis), maximum Cr and Ni contents normalized to all cation content in that layer (right axis) depending on temperature.

Figure 10 illustrates the temperature dependence of the average oxide film thickness, as well as Cr and Ni concentrations normalized to total cation concentration within the oxide. There is a certain correlation between thickness decrease from 160 to 180 °C and the increase in the concentration of Cr and to a lesser extent Ni, which can be traced to a larger amount of dissolved Fe from the oxide at that temperature leading to its substitution by Cr (and probably Ni) in the oxide, their dissolution rate being negligible face to Fe. Thus, even for an alloy initially containing only 0.6%Cr, the effect of this alloying element on flow-assisted corrosion seems to be already significant, as discussed in relevant reviews of the process [20,21,22,23].

Summarizing, the results from GDOES analysis indicate that in turbulent conditions, a single layer of oxide is formed on the 0.6% Cr steel, and Cr enrichment plays an essential role in determining the corrosion rate and the extent of film formation in such condition. An attempt to rationalize these findings is made in the Section 3.

## 3. Discussion

### 3.1. The Kinetic Model

Electrochemical impedance data are quantitatively interpreted by the mixed-conduction model for oxide films (MCM) [31,33,34]. For a corrosion film similar to magnetite containing Cr and to a lesser extent Ni (M_3_O_4_), the following reactions occur at the alloy–film (A/F) and film–solution (F/S) interfaces.

(1) $$\begin{array}{l} \mathrm{Film}\;\mathrm{growth}\;\mathrm{and}\;\mathrm{dissolution}\\ {(A/F)\;3M}_{m}\overset{k_{O}}{\to }{3M}_{M}{+4V}_{O}^{••}+8e^{\prime }\\ \left(F/S\right){\;4H}_{2}{O+4V}_{O}^{••}\overset{k_{2O}}{\to }{4O}_{O}{+8H}^{+}\\ {(F/S)\;M}_{3}O_{4}{+2H}^{+}+{2e}^{-}{+2H}_{2}O\overset{k_{d}}{⇄}{3M(\mathrm{OH})}_{2,\mathrm{aq}}\\ \mathrm{Iron}\;\mathrm{dissolution}\;\mathrm{through}\;\mathrm{the}\;\mathrm{film}\;(\mathrm{release})\\ {(A/F)\;\mathrm{Fe}}_{m}\overset{k_{Fe}}{\to }{\mathrm{Fe}}_{i}^{••}+2e^{\prime }\\ {(F/S)\;\mathrm{Fe}}_{i}^{••}\overset{k_{2\mathrm{Fe}}}{\to }{\mathrm{Fe}}_{\mathrm{aq}}^{y+}+(y-2)e^{\prime }\\ \mathrm{Coupled}\;\mathrm{reduction}\;\mathrm{of}\;\mathrm{water}\\ {(F/S)\;H}_{2}{O+2e}^{-}\to H_{2}+{2\mathrm{OH}}^{-} \end{array}$$

The rates of interfacial processes are expressed by the respective rate constants (*k*_i_, i = O, Fe, 2O, 2Fe) that depend on the potential drop at the respective interface in an exponential manner customary in electrochemical kinetics. The processes at the inner and outer interfaces are coupled by inward oxygen transport via vacancies and outward iron transport via interstitials. Ultimately, the above reactions lead to parallel generation of soluble iron via formation, transport and ejection of interstitial iron cations, and hydroxide particles at the film–solution interface via dissolution of the magnetite-type oxide. Accordingly, the transfer function that describes the impedance of the system has the form [31].

(2) $$Z=R_{el}+Z_{ox}+Z_{F/S},\;Z_{F/S}=\frac{1}{j\omega C_{F/S}+R_{F/S}^{-1}},\;Z_{f}={\left(Z_{e}^{-1}+Z_{ion,O}^{-1}++Z_{ion,Fe}^{-1}\right)}^{-1}$$

In the above equations, the impedance of the film–solution interface (*Z_F_*_/*S*_) is represented as a parallel combination of an interfacial capacitance (*C_F_*_/*S*_) and a charge transfer resistance of a single-step water reduction coupled to corrosion (*R_F_*_/_*_S_*). On the other hand, the impedance of the film *Z_f_* is a parallel combination of the impedances of its electric properties, *Z_e_*, and two ionic transport impedances corresponding to oxygen (*Z_ion_*_,*O*_) and iron (*Z_ion_*_,*Fe*_), respectively. The detailed expressions of these impedances are given below:

(3) $$Z_{e}\approx \frac{RT}{2j\omega FELC_{sc}}\ln\frac{\left[1+j\omega \rho_{d}\varepsilon \varepsilon_{0}\exp\left(2KL\right)\right]}{1+j\omega \rho_{d}\varepsilon \varepsilon_{0}},\;K=\frac{F}{RT}E,\rho_{d}=\frac{RT}{F^{2}D_{e}}\frac{k_{2O}+k_{2Fe}}{k_{O}+k_{Fe}}$$

(4) $$Z_{ion,O}\approx \frac{RT}{4F^{2}k_{O}(1-\alpha )\left(1+\sqrt{1+\frac{4j\omega }{D_{O}K^{2}}}\right)},\;Z_{ion,M}\approx \frac{RT}{4F^{2}k_{Fe}(1-\alpha )\left(1+\sqrt{1+\frac{4j\omega }{D_{Fe}K^{2}}}\right)}$$

Here, *C_sc_* is the space charge capacitance of the oxide, *D_e_*, *D_O_*, and *D_Fe_* are the diffusion coefficients of electronic carriers, oxygen vacancies, and cation interstitials, *L* is the film thickness, ***E*** is the electric field strength in the oxide, ε its dielectric constant (assumed to be 12 [33], and α is the polarizability of the oxide–coolant interface (taken as 0.9 [34]). The resistance of ionic transport can be calculated as the zero-frequency limit of the ionic transport impedances.

(5) $$R_{ion}=\frac{RT}{8F^{2}(1-\alpha )\left(k_{O}+k_{Fe}\right)}$$

Further, the film thickness increases with time following the equation derived previously [31].

(6) $$L=L_{0}+\frac{1}{b_{O}}\ln\left[1+V_{m,ox}k_{O}b_{O}e^{-b_{O}L_{0}}t\right],\;b_{O}=\frac{3\alpha_{O}FE}{RT}$$

In this equation, *L*_0_ is the initial oxide thickness (taken as 3 nm), *V_m_*_,*ox*_—the molar volume of the oxide (44.5 cm^3^ mol^−1^ for magnetite) and α_O_—the transfer coefficient of the oxidation reaction (assumed to be 0.1).

### 3.2. Parameterization

**Figure 11:**
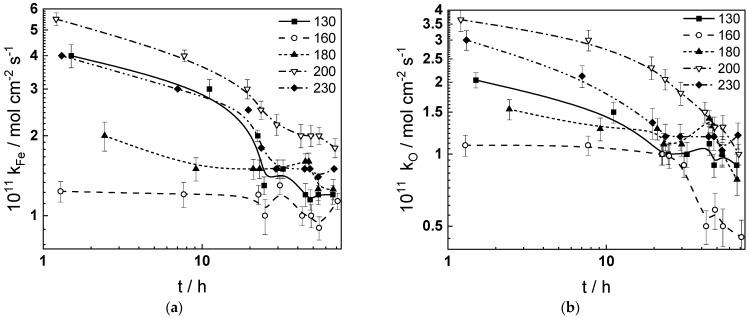
Dependence of the rate constants *k_M_* (**a**) and *k_O_* (**b**) at the alloy–film interface on time and temperature.

**Figure 12:**
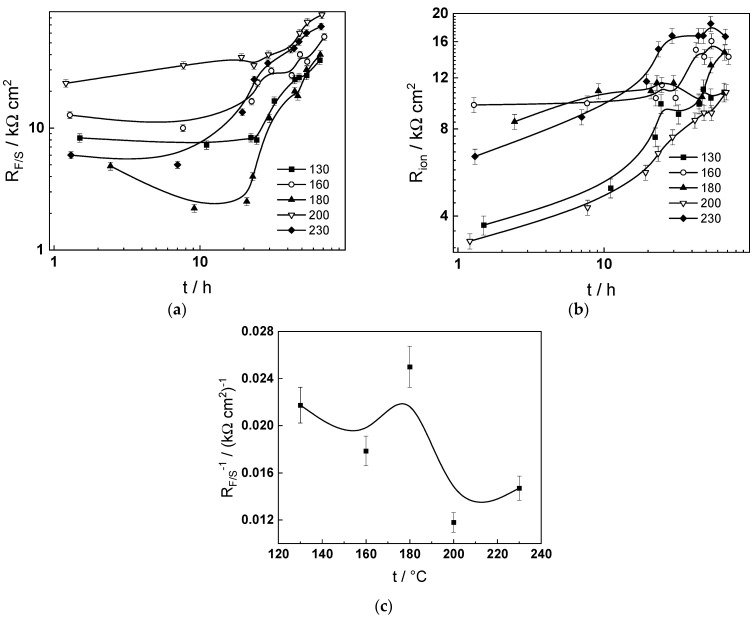
Dependence of the charge transfer resistance at the film–solution interface (**a**) and the ion transport resistance (**b**) on time and temperature; (**c**) dependence of the inverse of the charge transfer resistance (*R_F/S_^−^*^1^) after 72 h on temperature.

**Figure 13:**
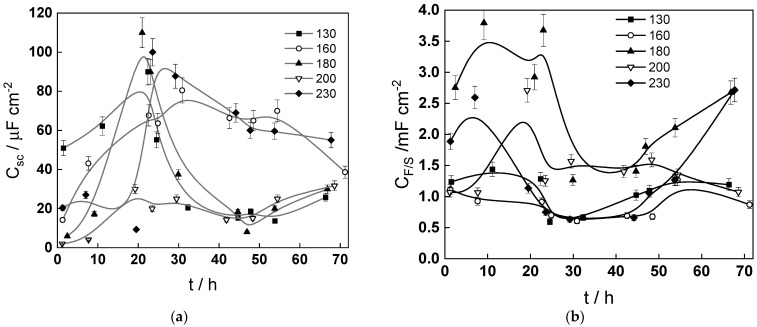
Dependence of the space charge capacitance (**a**) and the interfacial capacitance (**b**) on time and temperature.

**Figure 14:**
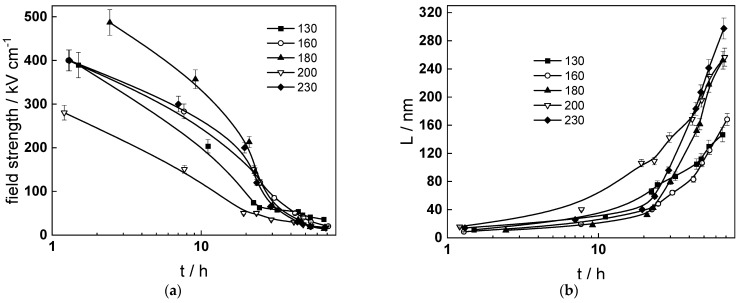
Dependence of field strength (**a**) and film thickness (**b**) on time and temperature.

Impedance spectra calculated via non-linear regression of experimental data with respect to the equations of the model are shown with solid lines in Figure 5, Figure 6 and Figure 7 and illustrate the ability of the model to account for both the magnitude and the frequency distribution of the experimental data within the reproducibility limit (±2% by magnitude and ±3° by phase shift). The dependences of the main parameters that depend on time are collected in Figure 11, Figure 12, Figure 13 and Figure 14, the respective errors of estimate (that did not exceed ±10%) being shown with vertical bars.

The rate constants of film growth and iron oxidation to form interstitials at the alloy–film interface (Figure 11a,b) decrease following the increase in flow rate at 24 h, which may seem counter-intuitive at first since this interface is not in direct contact with the solution. Taking into account the fact that the film growth and iron oxidation are balanced by film dissolution and iron cation ejection in the solution, the calculation result can be interpreted by an increase in both dissolution and cation ejection rates that lead to enrichment of the oxide with Cr (and to a lesser extent Ni, observed in the depth profiles). This enrichment would lead to dissolution rates slower than that of pure Fe oxide, hence the decrease in growth rate at the alloy–film interface.

The field strength in the oxide (Figure 14a) decreases with time and reaches constant values after ca. 50 h. The decrease is probably due to formation of space charge of point defects and substitute ions [35]. The corrosion film thickness (Figure 14b) follows a direct logarithmic law at all temperatures in agreement with model assumptions. The values at ca. 72 h are well comparable to the average values estimated from GDOES depth profiles. The increase in flow rate from 2 to 10 dm^3^ h^−1^ has certain influence on field strength, whereas the return to 2 dm^3^ h^−1^ has practically no effect. In other words, the oxide formed in turbulent conditions is stable with regard to hydrodynamic parameters, as already concluded on the basis of experimental impedance spectra.

On the other hand, the charge transfer resistance increases with flow rate, indicating that the electronic properties of the oxide have changed. This is corroborated by the significant increase in both space charge capacitance (assuming that the depletion layer is located at the film–solution interface) and the interfacial capacitance following the change in flow rate from 2 to 10 dm^3^ h^−1^. The change in the electronic properties of oxide at the interface seems to be to a great extent irreversible since the decreasing step in the flow rate at 48 h has a much weaker influence.

As a general picture, the influence of flow rate on corrosion of low-alloy steel seems to be transient in nature, the dissolution of Fe provoking an enrichment of Cr and accordingly a decrease in the rate of electronic charge transfer at the film–solution interface. In turn, this will lead to a decrease in oxide dissolution and cation ejection rates that ultimately will result in a lesser extent of corrosion.

From the point of view of recent reviews of flow-assisted corrosion [18,19,20], the most interesting dependence is that of the inverse of the charge transfer resistance at the oxide–coolant interface (*R_F/S_^−^*^1^, corresponding to the rate of interfacial electron transfer) on temperature (Figure 12c). A maximum is observed at 180 °C in accordance with data for flow-assisted corrosion rates in steam generators of both thermal and nuclear power plants. Thus, it can be hypothesized that the rate of flow-assisted corrosion in the studied conditions is determined by charge transfer at the corrosion film–coolant interface. In turn, the observed maximum is due to the superimposed decrease in magnetite solubility and increase in oxide dissolution rate with increasing temperature [22].

## 4. Materials and Methods

The experiments described in this paper are performed with a special flow-through cell made of AISI 316 stainless steel (Figure 1a), mounted immediately after the preheater laboratory made a recirculation loop. A flow-accelerating insert is located immediately before the cell. The working electrode is a cylindrical low-alloy steel sample that faces the accelerated flow, and the counter and reference electrodes are Pt and Pd, respectively. The Pd is polarized with a negative current of 10–30 µA to approximate the reversible hydrogen electrode. All the potentials in this paper are given in the standard hydrogen electrode (SHE) scale.

The k-ε turbulent flow model was implemented using a finite element method with commercial software (Comsol Multiphysics 6.1, Burlington, MA, USA) to simulate the hydrodynamics of pure water at 130–230 °C and 90 bars (density 0.95–0.82 g cm^−3^, dynamic viscosity 10^−3^–10^−4^ Pa s) at large Reynolds numbers.

The composition of the low-alloy steel (a commercially available welding rod) is listed in Table 1.

The electrolyte used was 0.1 mmol dm^−3^ MEA (pH_25 °C_ = 9.8) corresponding to typical concentrations used in steam generators to achieve a pH of ca. 10 [6,7]. To prepare the electrolyte, p.a. HOCH_2_CH_2_NH_2_ (Sigma Aldrich, St. Louis, MO, USA) with Fe content of less than 0.00005% and de-ionized water (conductivity less than 0.2 µS cm^−1^) were employed. Quantum chemical calculations of the MEA structure are performed with Arguslab 4.0.1 (Planaria Software LLC, Seattle, WA, USA).

Measurements were performed at 130, 160, 180, 200, and 230 °C, which mimic startup (up to 200 °C) and normal operation of a steam generator (at 230 °C). The following procedure was used:

(1) The loop is filled with electrolyte.
(2) The flow-through cell is heated up to 80 °C, compressed to 90 bars, and purged with N_2_ (99.999%) for 16 h to reach a dissolved oxygen concentration <10 µg kg^−1^. Purging of the feedwater in the reservoir with N_2_ continued for all the experiment duration (typically 72 h).
(3) After reaching this value, the temperature is increased to reach the highest measurement temperature (230 °C) for ca. 2 h.
(4) The inlet flow rate in the cell was 2 dm^3^ h^−1^ during the first 24 h, 10 dm^3^ h^−1^ from 24–48 h and again 2 dm^3^ h^−1^ from 48 to 72 h.

Solution conductivity was continuously monitored using a 912 conductometer (Metrohm, Herisau, Switzerland), pH via a 781 pH/ion-meter (Metrohm, Herisau, Switzerland), and dissolved oxygen content using an amperometric micro-sensor (AMT Analysenmesstechnik GmbH, Rostock, Germany). Before each experiment, the instruments were calibrated in the appropriate conductivity (0–100 µS cm^−1^), pH (4–10) and dissolved oxygen (0–100 mg kg^−1^) using certified electrolytes provided by the manufacturer.

A CompactStat.h10030 potentiostat operating in the floating mode (Ivium Technologies, Eindhoven, The Netherlands) was employed to measure impedance spectra in a frequency range of 0.1 mHz–11 kHz with an ac signal of 40 mV (rms) or 5 μA (rms). Measuring spectra with signal amplitudes between 10 and 40 mV ensured linearity, whereas the Kramers–Kronig compatibility test was applied to check causality. Spectra measured in potentiostatic and galvanostatic conditions were identical within the reproducibility limit (±1% by impedance magnitude and ±2° by phase shift). An Origin Pro platform (Originlab, Northampton, MA, USA) was employed for complex non-linear least squares fitting of data to the kinetic model equations by the Levenberg–Marquardt algorithm.

To estimate the oxide thickness and in-depth distribution of oxide and alloy constituents, GDOES was used with a GD750HR apparatus (Spectruma Analytik, Hof, Germany). Typical operating parameters were a primary voltage of 950 V, a current of 9 mA, and a pressure of 3 hPa. Calibration was based on certified reference materials chosen to cover the elements present in a wide range of steels in the relevant concentration ranges.

## 5. Conclusions

In situ EIS in the temperature interval 130–230 °C at 90 bar and inlet volume flow rates 2–10 dm^3^ h^−1^ is employed to study flow-assisted corrosion of low-alloy steel (0.6% Cr) in MEA solution, simulating steam generator conditions. The thickness and in-depth composition of the formed oxides are studied by GDOES analyses. Hydrodynamic calculations indicate that the developed flow-through cell with an accelerating insert ensures linear flow rates up to 2 m s^−1^ and Reynolds numbers above 10^4^, i.e., turbulent conditions are achieved at a volume flow rate of 10 dm^3^ h^−1^. At this flow rate, an increase in the dissolved iron concentration and a decrease in pH are deducted from water chemistry monitoring, indicating a transient increase in the rates of oxide dissolution and iron cation release. This leads to a modification of the surface oxide via enrichment of Cr and to a certain extent Ni, which provokes changes in the electronic properties of the layer, thus decreasing the rate of interfacial charge transfer that most probably controls the corrosion process. Further simulations using molecular dynamics are currently underway to quantify the adsorption of MEA on magnetite and related oxides, and they will be communicated in a companion paper.

## Figures and Tables

**Table 1:** Chemical composition of the low-alloy steel used in the present investigation (wt.%, balance Fe).

Element, wt.%	C	Cr	Mn	Ni	P	S	Si
nominal	0.17–0.24	0.5–0.7	0.1–0.3	≤0.12	≤0.035	≤0.035	0.17–0.37
Analyzed by GDOES	0.20 ± 0.02	0.60 ± 0.06	0.09 ± 0.01	0.10 ± 0.01	0.012	0.02	0.24 ± 0.024

## Data Availability

The data presented in this study are available on request from the corresponding author.
